# Preoperative promestriene for hysteroscopy: a randomized clinical trial

**DOI:** 10.1590/1806-9282.20231559

**Published:** 2024-07-19

**Authors:** Isabela Maciel Caetano, Agnaldo Lopes da Silva, Rivia Mara Lamaita, Bernardo Avila Maia, Eduardha Santos Temponi Barroso, Eduardo Batista Candido

**Affiliations:** 1 Universidade Federal de Minas Gerais, Hospital das Clínicas, Woman's Health Department – Belo Horizonte (MG), Brazil.; 2 Universidade Federal de Minas Gerais, Department of Gynecology and Obstetrics – Belo Horizonte (MG), Brazil.; 3 Hospital Felicio Rocho – Belo Horizonte (MG), Brazil.; 4 Universidade Federal de Minas Gerais – Belo Horizonte (MG), Brazil.

**Keywords:** Hysteroscopy, Hormone replacement therapy, Cervical erosion, Lacerations, Uterine perforation

## Abstract

**OBJECTIVE:**

Intraoperative complications of hysteroscopy, such as the creation of a false passage, cervix dilatation failure, and uterine perforation, may require suspension of the procedure. Some patients refuse a new procedure, which delays the diagnosis of a possible serious uterine pathology. For this reason, it is essential to develop strategies to increase the success rate of hysteroscopy. Some authors suggest preoperative use of topical estrogen for postmenopausal patients. This strategy is common in clinical practice, but studies demonstrating its effectiveness are scarce. The aim of this study was to evaluate the effect of cervical preparation with promestriene on the incidence of complications in postmenopausal women undergoing surgical hysteroscopy.

**METHODS:**

This is a double-blind clinical trial involving 37 postmenopausal patients undergoing surgical hysteroscopy. Participants used promestriene or placebo vaginally daily for 2 weeks and then twice a week for another 2 weeks until surgery.

**RESULTS:**

There were 2 out of 14 (14.3%) participants with complications in the promestriene group and 4 out of 23 (17.4%) participants in the placebo group (p=0.593). The complications were difficult cervical dilation, cervical laceration, and vaginal laceration.

**CONCLUSION:**

Cervical preparation with promestriene did not reduce intraoperative complications in postmenopausal patients undergoing surgical hysteroscopy.

## INTRODUCTION

Approximately 50% of hysteroscopy complications are related to difficult cervix dilation^1^. Postmenopausal status is a risk factor for cervical stenosis^2^. When the cervix is stenosed, there is an increased risk of false passage, cervix dilatation failure, cervical laceration, and uterine perforation^3^. A false passage, if not identified, will most likely lead to uterine perforation and its associated complications. Thus, when a false passage is created during cervical dilation, the procedure must be discontinued^4^.

In four studies that evaluated 554, 976, 5000, and 31052 patients undergoing office hysteroscopy, it was not possible to complete the procedure in 9.5, 8.9, 5.2, and 6.2% of patients, respectively. The four studies reported pain and cervical stenosis as the major causes of failure^5-8^. One of them reported cervical stenosis in 32.7% of the patients, most of them managed successfully (70.1% of them in postmenopausal women)^8^.

In a retrospective study of 516 patients undergoing office hysteroscopy, the authors described failure to access the uterine cavity in 62 patients (12%). Of these 62 patients, 36 refused to undergo a new procedure and 26 underwent a new procedure. All patients who underwent a new procedure had an anatomopathological diagnosis of an endometrial pathology, including endometrioid carcinoma and endometrial hyperplasia. Thus, it is extrapolated that an endometrial pathology may have been belatedly diagnosed in 36 patients who refused a new procedure. For this reason, the authors suggested that measures to increase the success rate of the first hysteroscopy be taken, such as for postmenopausal patients, the preoperative use of topical estrogen^9^.

Some of the strategies already studied are cervical preparation with misoprostol alone or associated with topical estrogen, as presented below. It must be highlighted that misoprostol in Brazil is only available in maternity hospitals.

A systematic review evaluated the use of misoprostol in cervical preparation for hysteroscopy and showed that intraoperative complications were less common in the misoprostol group than in the placebo group. There was a significant reduction in the incidence of cervical laceration and creation of a false passage, but only for patients in their reproductive years. This procedure was not beneficial for postmenopausal women. Side effects were observed in 24 out of 136 (19%) participants of the misoprostol group and 12 out of 136 (9%) participants of the control group. Misoprostol was associated with mild abdominal pain, increased body temperature, and vaginal bleeding. There was no conclusive evidence regarding nausea, shivering, or diarrhea^1^.

A randomized clinical trial compared the use of estradiol alone with the use of estradiol plus misoprostol in cervical preparation for hysteroscopy in postmenopausal patients. Patients were divided into two groups: (a) those who used topical estrogen for 14 days before hysteroscopy and misoprostol 12 h before surgery and (b) those who used topical estrogen for 14 days before hysteroscopy and placebo 12 h before surgery. Group (a) had significantly better cervical ripening compared to group (b). There was one uterine perforation in each group. There was a significant difference between the initial measurement of the diameter of the external orifice of the cervix in the office evaluation before the medications and the measurement at the time of hysteroscopy, in both groups: in group (a), from 2.6 mm to 5.7 mm, and in group (b), from 2.1 mm to 4.7 mm. There was no control group. They found that estradiol alone could increase the diameter of the external orifice of the cervix, but it is not known if it would imply a clinically significant outcome (i.e., reduction of intraoperative complications)^10^.

There exists a gap in the medical literature on the use of isolated topical estrogen for cervical preparation for hysteroscopy. Conversely, it is common in clinical practice.

Topical estrogen plays an important role in the treatment of genitourinary syndrome of menopause^11^. The improvement of urogenital symptoms and cytology usually occurs after 5–14 days of local therapy^12^. Estriol, estradiol, and conjugated estrogen, even when applied vaginally, reduce FSH. Promestriene does not reach the systemic circulation, cannot be converted to estradiol, does not alter the serum level of gonadotropins and estradiol, and does not stimulate the endometrium. Its systemic inactivity justifies its use when active estrogens are contraindicated, as in patients with estrogen-sensitive cancers^12^. Studies have already evaluated the efficacy and safety of promestriene in such patients^12-15^.

The aim of this study was to evaluate the effect of promestriene on the incidence of intraoperative complications in postmenopausal women undergoing surgical hysteroscopy.

## METHODS

The study was approved by the ethics committee of the research center and by Plataforma Brasil of Brazil's National Health Department (CAAE 38240720.0.0000.5123, deliberation number 4.508.539 and 4.984.613). It is registered in The Brazilian Registry of Clinical Trials (ReBEC) and is reported according to the CONSORT guidelines.

The research center of this randomized clinical trial was a tertiary hospital in Belo Horizonte, Brazil. This is a pilot study so the sample size was not previously determined. Over a 12-month period, postmenopausal patients who met the criteria were invited to participate in the trial. The promestriene or placebo and vaginal applicators were placed in numbered opaque envelopes, according to the randomization. Randomization was made in Microsoft Excel^Ò^ in blocks of 50 and in a 1:1 ratio. The envelopes were packed and sealed by a physician who did not meet the participants. Then, the envelopes were delivered to the participants by one of the authors, along with the consent form and written instructions. Participants were supposed to use the vaginal cream (promestriene or placebo) once a day for 2 weeks and, after that period, twice a week for another 2 weeks, until the procedure was done.

The placebos and their vaginal applicators were funded by the researchers. All the promestriene needed for the study were provided by Eurofarma^Ò^. There was no transfer of cash.

**Figure 1:**
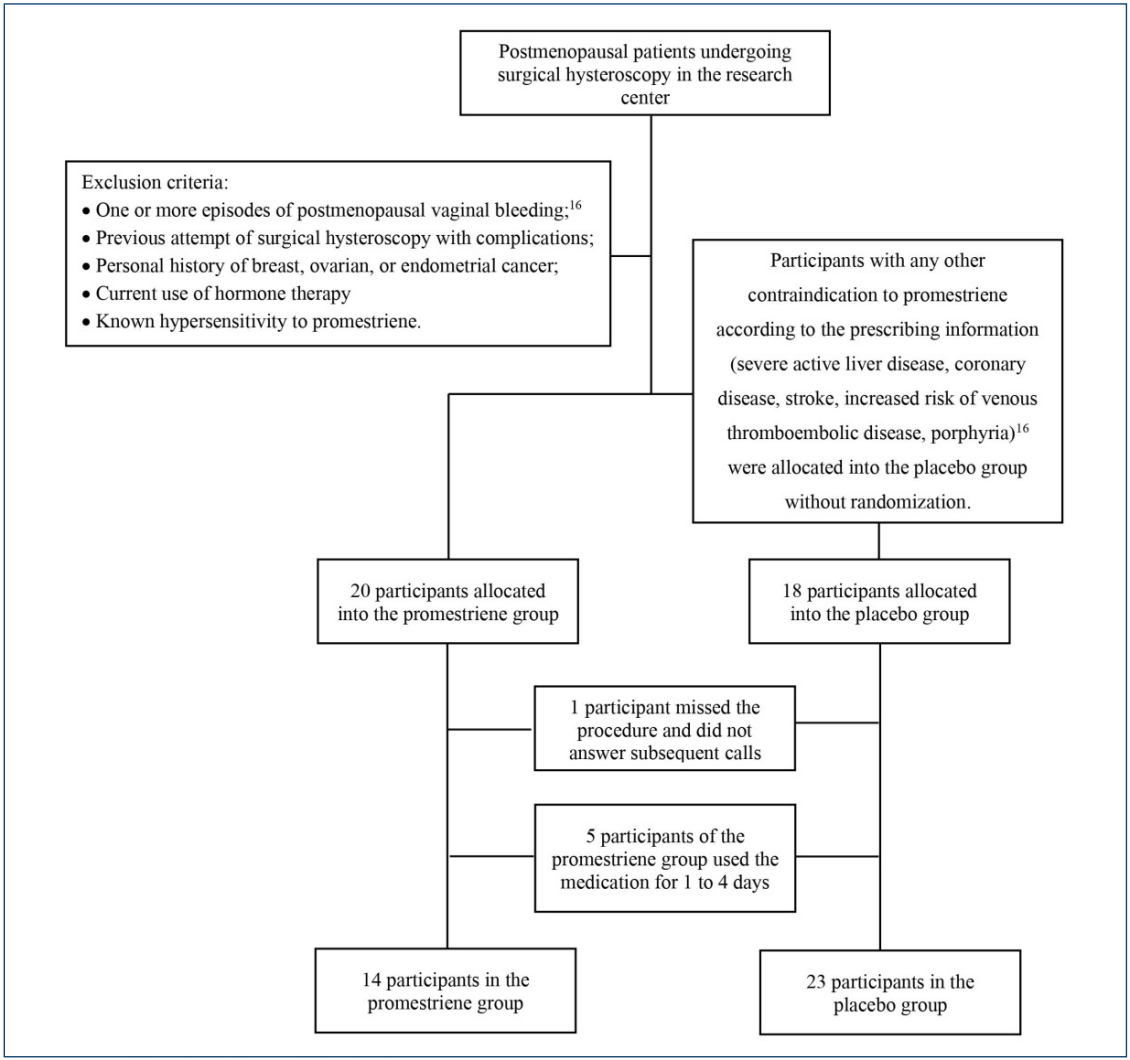
Participants' flowchart.

Participants' flowchart with inclusion and exclusion criteria are described in Figure 1. The shortage of supplies and the suspension of elective procedures due to the COVID-19 pandemic limited the number of participants.

All procedures were performed by the same team with a Kalz Stoltz 27050 SL outer sheath and a 4 mm/30º telescope. The surgeons did not know which group each participant belonged to. The participants were asked over telephone about the use of the vaginal cream in the postoperative appointment or later. The authors read the participants' operative report and noted any described complications.

Statistical analysis was conducted to describe the baseline characteristics of the study participants. For continuous variables, the mean, standard deviation, median, quartiles, and minimum and maximum values were used. For categorical variables, the absolute and relative frequencies were described. In the evaluation of categorical variables, the authors used the chi-square and Fisher's exact tests. The Mann–Whitney test was used to compare continuous variables between groups since the variables did not show normal distribution by the Shapiro-Wilk test. All analyses were performed using the Stata software, version 16 and a 5% significance level was considered.

## RESULTS

Participants were enrolled from August 2021 to July 2022. Data analysis of baseline characteristics is described in Table 1.

**Table 1:** Participants' baseline characteristics.

Characteristics		Promestriene group Mean (±SD)	Placebo group Mean (±SD)	p-value
Body weight		69.2 (±9.6)	70.2 (±13.7)	0.820
BMI		26.4 (±3.7)	27.3 (±5.1)	0.805
Number of pregnancies		2.9 (±2.2)	3.1 (±2.0)	0.423
Number of vaginal deliveries		1.8 (±2.2)	2.3 (±2.2)	0.404
Number of cesarian sections		0.6 (±0.8)	0.6 (±1.1)	0.664
Number of pregnancy losses		0.5 (±0.7)	0.2 (±0.7)	0.094
Menopause		50.5 (±3.2)	48.8 (±4.6)	0.291
Duration of medication use		30.6 (±1.7)	–	–
Age at surgery		59.4 (±5.8)	64.8 (±6.6)	0.019
Ethnicity	White	7	12	0.999
	Pardo	4	8	
	Black	2	3	
Educational qualification	Middle school	7	15	0.693
	High school	5	6	
	Bachelor's degree	1	1	

Exactly five participants were reallocated to the placebo group because they used promestriene for only 0 to 4 times. Two participants reported that they discontinued after using it three and four times due to discomfort associated with the applicator, while the other three reported using it 0, 1, and 3 times because they forgot to use it. No participant had any serious adverse event to the medications.

Of the 37 participants, 6 (16.21%) experienced complications, all of which were minor (Table 2). No complications resulted in the suspension of the procedure, prolonged hospital stay, or involved laparotomy. The participants who experienced complications were as follows:

- Promestriene group, 54 years old, cervical laceration requiring suture
- Promestriene group, 65 years old, cervical laceration not requiring suture
- Placebo group, 71 years old, difficult dilation
- Placebo group, 71 years old, difficult dilation
- Placebo group, 71 years old, difficult dilation and vag
- inal laceration requiring suture
- Placebo group, 74 years old, cervical laceration requiring suture

**Table 2:** Incidence of complications.

Complications		Promestriene group		Placebo group		p-value
		n	%	n	%	
Number of participants with laceration of the cervix or vagina	Yes	2/14	14.3	2/23	8.7	0.491
	No	12/14	85.7	21/23	91.3	
Number of participants with difficulty in cervical dilation	Yes	0/14	0	3/23	13	0.228
	No	14/14	100	20/23	87	
Number of participants with any complication	Yes	2/14	14.3	4/23	17.4	0.593
	No	12/14	85.7	19/23	82.6	

In the placebo group, participants with complications were significantly older (mean age 72.0 years; SD ±1.4) than participants who did not have experience complications (mean age 63.3 years; SD ±6.3) (p=0.005). There was no age difference observed between patients who had complications and patients who did not in the promestriene group.

## DISCUSSION

Until August 2023, there were no studies in Portuguese or English evaluating topical estrogen versus placebo or control group for preparation for hysteroscopy. This is a gap in medical literature, even though it is common in clinical practice.

The hypothesis that topical estrogen alone reduces complications in postmenopausal patients is biologically plausible, as shown by a study that demonstrated that the use of topical estrogen increases the diameter of the external orifice of the cervix of postmenopausal women^10^.

There is a concern about prescribing promestriene to patients with endometrial thickening due to the possibility of endometrial malignancy. However, the literature review found studies that demonstrate the safety of promestriene in patients with estrogen-dependent cancer^12-15^. The drug was used by the participants for 4 weeks, which is the usual (4–6 weeks) time of use in preparation for prolapse correction surgeries^17^.

There was a significant difference between the groups in terms of age. This difference was attributed to the fact that patients with contraindications to hormone therapy were placed into the placebo group without randomization (i.e., all patients with coronary disease, stroke, and high risk of venous thromboembolic disease). As these diseases become increasingly prevalent with aging, patients with these diseases are expected to be older.

There was no significant difference between the groups in terms of number of participants with any complication, number of participants with cervix/vaginal laceration, and number of participants with difficulty in cervical dilation.

In the placebo group, participants who experienced complications were significantly older than those who did not experience complications. This finding is consistent with the literature: a higher rate of complications in hysteroscopy is expected in patients with hypoestrogenism^8^. On the contrary, in the promestriene group, there was no significant difference in terms of age between the participants who had complications and the participants who did not. Once genital atrophy had been treated, age ceased to be a risk factor for complications, which is a remarkable finding.

The sample size of this study was a limiting factor. Considering that hysteroscopy is a procedure with a low rate of complications, a large number of participants are necessary to show any significant difference.

## CONCLUSION

Cervical preparation with promestriene did not demonstrate a reduction in the incidence of intraoperative complications in postmenopausal patients undergoing surgical hysteroscopy.

## ETHICAL APPROVAL

The study was approved by the ethics committee of the research center and by Plataforma Brasil of Brazil's National Health Department on January 21, 2021 (CAAE 38240720.0.0000.5123, deliberation number 4.508.539 and 4.984.613. It is registered in The Brazilian Registry of Clinical Trials (ReBEC).
